# Challenges of using generative artificial intelligence for diabetes patient education: a cross-platform analysis of the quality, readability, and actionability of text generated by large language models

**DOI:** 10.3389/fpubh.2026.1804524

**Published:** 2026-03-30

**Authors:** Zhiqiang Wang, Xiaoya Li, Xianglan Tao, Jie Li, Li Zhang, Xiaorong He, Jing Yang

**Affiliations:** 1 Guangyuan Central Hospital, Guangyuan, Sichuan, China; 2 Medical School of Yangtze University, Jingzhou, Hubei, China

**Keywords:** cross-platform evaluation, diabetes mellitus, diabetes self-management education and support (DSMES), large language model (LLM), readability, text quality

## Abstract

**Objective:**

To compare, across large language model (LLM) platforms, the quality, readability, and completeness of action-oriented instructions in diabetes self-management education texts, and to quantify the associations among these domains to inform model selection and risk mitigation.

**Methods:**

Ten LLM platforms were used to generate diabetes education texts (total *n* = 200), stratified by topic. Outcomes included the Global Quality Score (GQS), the Patient Education Materials Assessment Tool for Printable Materials (PEMAT-P), and EQIP-36 (Ensuring Quality Information for Patients, 36-item version). Text characteristics, including word count, sentence count, and syllable count, were recorded. Readability was assessed using the Automated Readability Index (ARI), Coleman–Liau Index (CLI), Flesch–Kincaid Grade Level (FKGL), Flesch Reading Ease Score (FRES), Gunning Fog Index (GFOG), Linsear Write (LW), and the Simple Measure of Gobbledygook (SMOG). Between-platform differences were evaluated using one-way ANOVA or the Kruskal–Wallis test, as appropriate. Associations between readability indices and GQS, PEMAT-P, and EQIP-36 were examined using correlation heat maps and exploratory stepwise multiple linear regression. Because the readability indices were highly intercorrelated, these regression analyses were considered exploratory and were used to identify candidate readability-related correlates rather than definitive independent predictors.

**Results:**

GQS and PEMAT-P differed significantly across platforms (both *p* < 0.001), whereas EQIP-36 did not (*p* = 0.062). Text length and readability also varied by platform (most *p* < 0.001). After stratification by topic, PEMAT-P understandability, PEMAT-P total score, and GQS no longer differed significantly across topics (*p* = 0.356, *p* = 0.247, and *p* = 0.182, respectively), whereas PEMAT-P actionability (*p* < 0.001), EQIP-36 (*p* < 0.001), and several readability metrics remained significantly different. Difficulty indices were strongly intercorrelated, and FRES was inversely associated with multiple difficulty indices. Exploratory regression analyses suggested that greater reading burden tended to co-occur with lower GQS, PEMAT-P, and EQIP-36 scores.

**Conclusion:**

LLM-generated diabetes education texts exhibit marked cross-platform heterogeneity, and exploratory analyses suggest a potential trade-off between readability and both information quality and the completeness of action-oriented instructions. Clinical implementation should therefore combine careful platform selection, structured prompting with templates, human–AI review, and continuous quality monitoring to support safe, readable, and actionable patient education.

## Introduction

The burden of diabetes continues to rise, posing a major challenge to global public health and health-care systems (1). Recent estimates suggest that more than 800 million people worldwide are living with diabetes (1). Because effective diabetes care depends heavily on sustained self-management, structured diabetes self-management education and support (DSMES) has been shown to improve metabolic control and reduce the risk of diabetes-related adverse outcomes (2, 3). In real-world practise, however, access to DSMES remains limited because of workforce shortages, geographical barriers, and time-constrained clinical encounters (2, 4). Consequently, patients are increasingly turning to online sources for self-management advice. In the United States, approximately 74% of adults use the internet, and nearly 80% report having searched online for health information (5). Yet online health information often exceeds recommended literacy thresholds (6–8), disproportionately burdening disadvantaged populations and limiting their ability to interpret and implement essential self-management practises. Improving the accessibility and readability of diabetes education materials—so that guidance is both understandable and actionable—has therefore become an urgent implementation priority in digital health.

In this rapidly evolving landscape, artificial intelligence (AI), particularly large language models (LLMs), has emerged as a potentially valuable tool for diabetes education (9, 10). LLMs can generate sophisticated natural-language responses and engage interactively with patients, potentially helping to address workforce, geographical, and time-related constraints in the delivery of education (11–14). Previous studies in health-care question-answering settings have shown that ChatGPT-generated patient-facing responses may perform acceptably in terms of accuracy, informational breadth, and conciseness, and have been rated favourably in selected evaluations (15, 16). Nevertheless, the usefulness of LLMs in patient education ultimately depends on whether their outputs are consistently readable, trustworthy, and responsive to patients’ needs and care contexts; systematic evaluation is therefore essential.

Despite their fluency, educational texts generated by LLMs may not align with the reading abilities of the general public. Such outputs may be overly long, densely laden with technical terminology, and generated without effective constraints, resulting in readability levels that exceed recommended standards (8, 15). Although simplification may improve readability, it may do so at the expense of completeness; one review found that simplification could omit up to 83% of important content (17), thereby compromising usability and the provision of actionable guidance. For conditions such as diabetes, which require daily self-management, the absence of clear, stepwise, skills-based instructions may substantially diminish educational effectiveness. Rather than accepting a trade-off between readability and completeness, optimisation should aim to preserve factual accuracy, actionability, and informational completeness simultaneously.

Patient education also demands a high degree of accuracy and reliability, because errors in recommendations may pose direct health risks. General-purpose LLMs remain susceptible to hallucinations—apparently credible statements that lack adequate supporting evidence (18–23). Moreover, outputs delivered in an authoritative tone may mislead non-expert users (22). Accordingly, the World Health Organisation (WHO) has urged caution regarding the use of generative AI in health care until its safety and effectiveness have been adequately established (22). Before LLMs can be applied to diabetes self-management education, systematic pre-implementation assessment and validation are therefore required, together with safeguards that anchor outputs to authoritative sources to reduce the risk of error. In addition, customised medical LLMs and chatbots are evolving rapidly, and platform-level styles and priorities differ substantially, underscoring the need for cross-platform comparison and standardised evaluation.

Against this background, the present study compares the performance of LLM platforms in diabetes self-management education with respect to readability, credibility, comprehensibility, and actionability, and examines potential trade-offs among overall quality, readability, and the completeness of action-oriented instructions. We combined seven readability indices—the Automated Readability Index (ARI), Coleman–Liau Index (CLI), Flesch–Kincaid Grade Level (FKGL), Flesch Reading Ease Score (FRES), Gunning Fog Index (GFOG), Linsear Write Formula (LW), and Simple Measure of Gobbledygook (SMOG)—with the Global Quality Score (GQS), Patient Education Materials Assessment Tool for Printable Materials (PEMAT-P), and Ensuring Quality Information for Patients (EQIP-36) to evaluate model-generated materials. Ten representative models were assessed: ChatGPT 4o; ChatGPT 5.1-Thinking; ChatGPT 5.2-Thinking; DeepSeek-R1; Doubao; ERNIE Bot 5.0 Preview; Kimi K2; Qwen3; Qwen3-Max; and Qwen3-Max-Thinking-Preview. This cross-platform design enhances comparability, delineates platform-specific patterns in patient-education outputs, and provides a reusable framework to inform optimisation and risk governance in LLM-enabled digital health education.

## Materials and methods

### Study design and question collection

To construct the dataset, we conducted a targeted search of topic-specific literature and authoritative guidelines and, with input from Australian domain specialists, compiled an initial list of 20 frequently asked questions. The questions covered five domains: Fundamental Understanding of the Disease, Diagnosis and Classification, Treatment and Technology, Daily Management and Prevention, and Complications, Psychosocial Care, and Prognosis. The item development process was informed by previous similar studies to enhance clinical applicability (24, 25).

The consolidated question list was reviewed and approved by three endocrinologists, each with more than 10 years of clinical experience, to ensure content validity and representativeness. Discrepancies were resolved through a consensus process, yielding the final dataset. This systematic, multi-step approach produced a rigorously curated set of questions that formed the basis for subsequent evaluation of the quality, readability, actionability, and reliability of LLM-generated responses (see Table 1).

**Table 1:** List of frequently asked questions about diabetes.

Issue list
Fundamental understanding of the disease
1. What type of disease is diabetes mellitus?
2. What is the core pathophysiologic mechanism of type 2 diabetes?
3. What is the most common type of diabetes mellitus in adults?
4. Does type 2 diabetes have a genetic predisposition?
Diagnosis and classification
1. Which laboratory biomarker is most commonly used to diagnose diabetes mellitus?
2. What HbA1c level is diagnostic of diabetes mellitus?
3. How is diabetes mellitus etiologically classified in clinical practise?
4. How is hypoglycemia classified into Levels 1–3 in the ADA Standards of Care?
Treatment and technology
1. Which medication is commonly recommended as initial pharmacologic therapy for most adults with type 2 diabetes when not contraindicated?
2. What is the primary therapeutic role of SGLT2 inhibitors in people with type 2 diabetes and chronic kidney disease?
3. What is the primary therapeutic role of GLP-1 receptor agonists in people with type 2 diabetes and obesity?
4. Under what circumstances should insulin be selected as the first injectable therapy in type 2 diabetes?
Daily management and prevention
1. Which macronutrient has the greatest immediate impact on postprandial blood glucose?
2. What is the main clinical purpose of continuous glucose monitoring in insulin-treated diabetes?
3. What is the “15–15 rule” for treating hypoglycemia in a conscious patient?
4. What is the purpose of daily foot inspection in people with diabetes?
Complications, psychosocial care, and prognosis
1. Which test is recommended for screening albuminuria in diabetic kidney disease?
2. Which examination is recommended for screening diabetic retinopathy?
3. What is diabetes distress?
4. When should HbA1c targets be set less stringently to prioritise safety over tight glycemic control?

### AI platform prompting and data collection

We conducted a cross-sectional study including representative large language models (LLMs) from 10 domestic and international conversational artificial intelligence (AI) platforms: ChatGPT 4o (OpenAI, 2024-05-13; multimodal), ChatGPT 5.1-Thinking (OpenAI, 2025-11-12; reasoning and tool use), ChatGPT 5.2-Thinking (OpenAI, 2025-12-11; advanced reasoning and extended-context capability), DeepSeek-R1 (DeepSeek, 2025-01-20; reasoning-optimised), Doubao (ByteDance, 2025-06-11; China-developed platform), ERNIE Bot 5.0 Preview (Baidu, 2025-06-30; knowledge-enhanced), Kimi K2 [Moonshot AI, 2025-07-11; open-source mixture-of-experts (MoE)], Qwen3 (Alibaba Cloud, 2025-04-28; general-purpose model), Qwen3-Max (Alibaba Cloud, 2025-04-28; high-performance variant), and Qwen3-Max-Thinking-Preview (Alibaba Cloud, 2025-06-21; enhanced reasoning). To ensure consistency in response language, all prompts were delivered in English.

For each prespecified question, we initiated a new, independent chat session on each platform and cleared the chat history before each query to minimise contextual carryover. This procedure follows common experimental practise in LLM evaluation, whereby each question is posed in a fresh session without prior contextual interference to facilitate independent and comparable outputs across platforms. During a single standardised collection window on Jan 2, 2026, we submitted 20 questions to each platform and recorded basic interaction metrics in real time, including response word and sentence counts. All AI-generated responses were retained verbatim, without manual editing, for subsequent evaluation and analysis.

### Evaluation instruments and metrics

To comprehensively assess the outputs of the 10 LLMs—ChatGPT 4o, ChatGPT 5.1-Thinking, ChatGPT 5.2-Thinking, DeepSeek-R1, Doubao, ERNIE Bot 5.0 Preview, Kimi K2, Qwen3, Qwen3-Max, and Qwen3-Max-Thinking-Preview—we applied three categories of evaluation instruments and metrics. These measures captured key dimensions of patient-oriented diabetes information, including understandability and actionability, overall information quality, and text readability.

All AI-generated responses were exported in a standardised format, stripped of platform and model identifiers, and assigned random codes before evaluation to minimise potential assessor bias arising from prior knowledge of the source model. Two researchers with experience in clinical assessment and health communication independently evaluated all responses using the PEMAT-P, EQIP-36, and GQS instruments. The 2 reviewers completed the initial scoring independently and were blinded to each other’s ratings. Prior to formal scoring, both evaluators received standardised training and completed pilot assessments on a prespecified sample to calibrate the scoring criteria. In the event of disagreement, the 2 reviewers first discussed the rating to reach consensus; if consensus could not be achieved, a third senior expert in diabetes care and patient education served as the adjudicator. Inter-rater reliability between the 2 primary reviewers was quantified using Cohen’s kappa coefficient, calculated from pre-adjudication agreement between the 2 primary reviewers, and was 0.91, indicating excellent agreement.

Specifically, PEMAT-P was used to assess the understandability and actionability of patient-facing materials, thereby quantifying whether readers could understand the content and identify concrete steps for diabetes self-management. EQIP-36 was used to evaluate whether key informational elements were presented in a complete and standardised manner, with particular emphasis on content structure and completeness beyond readability alone. GQS was used to provide an overall user-oriented assessment of global quality and perceived usefulness, thereby complementing the more structured checklist-based instruments.

In addition, multiple readability indices were used to quantify linguistic difficulty and objectively characterise text complexity. This step was important because actionability and information completeness may involve trade-offs with readability, and no single metric can fully capture these interrelated characteristics. As detailed in the following subsections, these instruments jointly enabled the evaluation of comprehension demands, overall information quality, and reading difficulty. For statistical analyses, the final score for each response was the consensus score established after independent rating and adjudication.

### Understandability and actionability assessment

The Patient Education Materials Assessment Tool for Printable Materials (PEMAT-P) was used to assess the understandability and actionability of patient education materials (26). Higher PEMAT-P scores indicate that a text is easier to understand and better supports readers in taking actionable steps (26). PEMAT-P scoring was performed in accordance with the original user guide. Item-level ratings were converted into domain-specific percentage scores for understandability and actionability, and an overall PEMAT-P score was calculated for comparative analyses.

### Quality assessment

The Ensuring Quality Information for Patients instrument (EQIP-36) is a 36-item scale used to assess the quality of information provided to patients. It comprises three domains—content, source identification/attribution, and structure—and evaluates the completeness, accuracy, and appropriateness of information presentation. The summed score reflects overall information quality and was reported as a total quality score ranging from 0 to 100 (27). EQIP-36 has been used to evaluate online health information and has demonstrated good reliability and validity in previous studies (28).

The Global Quality Score (GQS) is a general quality measure in which raters assign a score from 1 (very poor quality) to 5 (very high quality) to provide a subjective assessment of the overall usefulness and reliability of each response (29). Because GQS includes a subjective global judgement component, blinding and independent scoring were implemented to reduce potential assessor bias.

### Readability metrics

Readability was calculated for each response using seven established English readability formulas: the Automated Readability Index (ARI), Coleman–Liau Index (CLI), Flesch–Kincaid Grade Level (FKGL), Flesch Reading Ease Score (FRES), Gunning Fog Index (GFOG), Linsear Write Formula, and the Simple Measure of Gobbledygook (SMOG) (30–35). These indices estimate reading difficulty on the basis of word- and sentence-level features and differ in their scoring ranges and interpretation. With the exception of FRES, the other six indices correspond to US grade levels, with higher scores indicating that a higher level of educational attainment is required and that the text is more complex. By contrast, FRES ranges from 0 to 100, with higher scores indicating easier readability. To standardise computation, we used two online tools—ReadabilityFormulas.com (Calculator 1) and Online-Utility.org (Calculator 2)—which implement different readability formulas. Readability analysis enabled objective comparison of linguistic complexity across LLM-generated responses and, when combined with subjective ratings, helped determine whether the content was appropriate for comprehension by the general public.

### Statistical analysis

Because most variables were not normally distributed, non-parametric methods were used for the primary analyses. Continuous variables are presented as median (M) with interquartile range (IQR), expressed as M (Q1, Q3) or M (IQR). Differences between two groups were assessed using the Mann–Whitney U test, whereas comparisons across three or more groups were performed using the Kruskal–Wallis H test, followed by Dunn–Bonferroni post-hoc pairwise comparisons when the omnibus test was significant. Effect sizes were reported as r for two-group comparisons (*r* = *Z*/√*N*) and as epsilon squared (ε^2^), or an equivalent non-parametric measure, for comparisons involving multiple groups. Associations between variables were assessed using Spearman’s rank correlation coefficient. All tests were two-sided, and *p* < 0.05 was considered statistically significant.

For variables that met the assumptions of normality and homoscedasticity and were summarised as mean ± standard deviation, one-way analysis of variance (ANOVA) was used. All analyses were conducted using IBM SPSS Statistics version 25, and figures were generated using GraphPad Prism version 9.

To further explore the associations between readability and the quality of patient education materials, exploratory stepwise multiple linear regression analyses were performed using separate models with the PEMAT-P total score, EQIP-36 total score, and GQS as dependent variables. The seven readability indices—ARI, FRES, GFOG, FKGL, CLI, SMOG, and LW—were entered as candidate variables. Because these readability indices are conceptually related and may exhibit substantial multicollinearity, the regression analyses were intended as exploratory rather than confirmatory. Therefore, the variables retained by the exploratory models should be interpreted as candidate correlates of quality outcomes rather than definitive independent determinants. We report regression coefficients, *R*^2^/adjusted *R*^2^, the overall F test, the Durbin–Watson statistic, and collinearity diagnostics, including the variance inflation factor (VIF) and tolerance.

## Results

### Text characteristics

#### Characteristics of diabetes education texts generated by different large language model platforms

Among the 200 DSMES texts, clear platform-level differences were observed in both quality and readability outcomes (Tables 2, 3). The PEMAT-P understandability and total scores differed significantly across platforms (χ^2^ = 66.58 and 41.77, respectively; both *p* < 0.001), whereas the PEMAT-P actionability score remained uniformly low and showed no significant between-platform difference (*p* = 0.313). The EQIP-36 total score did not differ significantly across platforms (*p* = 0.062), but the GQS showed a significant between-platform difference (*χ*^2^ = 33.36, *p* < 0.001). Text length also varied significantly by platform, including word count, sentence count, and syllable count (all *p* < 0.001). Among the readability metrics, ARI, FRES, GFOG, FKGL, CLI, SMOG, and LW all differed significantly across platforms (all *p* ≤ 0.003). Overall, Qwen3 and DeepSeek-R1 showed relatively higher PEMAT-P understandability, DeepSeek-R1 achieved the highest GQS, Qwen3 generated the longest responses, and ChatGPT 5.1-Thinking demonstrated comparatively more favourable readability.

**Table 2:** Quality indicators of diabetes education texts generated across large language model platforms.

Variable	Total (*n* = 200)	ChatGPT 4o (*n* = 20)	ChatGPT 5.1-Thinking (*n* = 20)	ChatGPT 5.2-Thinking (*n* = 20)	DeepSeek-R1 (*n* = 20)	Doubao (*n* = 20)	ERNIE Bot 5.0 Preview (*n* = 20)	Kimi K2 (*n* = 20)	Qwen3 (*n* = 20)	Qwen3-Max (*n* = 20)	Qwen3-Max-Thinking-Preview (*n* = 20)	*p* value
PEMAT-P understandability	76.90 (66.70, 84.60)	59.90 (53.80, 66.70)	75.00 (66.10, 84.60)	66.70 (54.20, 76.90)	80.10 (76.90, 92.30)	76.90 (66.70, 88.70)	66.70 (58.30, 75.47)	76.90 (69.20, 86.90)	84.60 (76.90, 92.30)	76.90 (69.20, 80.83)	76.90 (76.90, 84.60)	< 0.001
PEMAT-P actionability	20.00 (0.00, 40.00)	20.00 (0.00, 21.25)	20.00 (0.00, 52.50)	10.00 (0.00, 40.00)	10.00 (0.00, 40.00)	8.35 (0.00, 20.00)	10.00 (0.00, 25.00)	10.00 (0.00, 20.00)	40.00 (0.00, 60.00)	0.00 (0.00, 20.00)	0.00 (0.00, 25.00)	0.313
PEMAT-P total score	58.80 (50.00, 68.20)	44.40 (43.15, 56.92)	61.10 (50.00, 77.80)	54.25 (40.90, 62.50)	61.10 (58.00, 73.60)	57.20 (52.17, 68.20)	52.90 (46.43, 59.37)	59.95 (55.60, 72.20)	70.20 (65.83, 78.67)	58.80 (52.17, 61.10)	61.10 (55.60, 67.88)	< 0.001
EQIP-36 score	38.20 (32.90, 43.90)	35.92 (29.70, 38.70)	37.47 (29.75, 43.82)	35.41 (25.43, 42.40)	41.81 (37.40, 45.59)	40.00 (36.01, 44.57)	37.32 (34.70, 44.40)	38.40 (35.22, 41.49)	42.78 (34.12, 47.86)	36.25 (34.38, 38.85)	35.23 (32.17, 39.78)	0.062
GQS score	4.00 (4.00, 4.00)	4.00 (4.00, 4.00)	4.00 (4.00, 5.00)	4.00 (4.00, 5.00)	5.00 (4.00, 5.00)	4.00 (4.00, 4.00)	4.00 (4.00, 4.00)	4.00 (4.00, 4.00)	4.00 (3.75, 4.00)	4.00 (3.00, 4.00)	4.00 (4.00, 4.00)	< 0.001

Values are presented as median (Q1, Q3). Omnibus p values were obtained using the Kruskal–Wallis H test. PEMAT-P, Patient Education Materials Assessment Tool for Printable Materials; EQIP-36, Ensuring Quality Information for Patients, 36-item instrument; GQS, Global Quality Score.

**Table 3:** Text length and readability indicators of diabetes education texts generated across large language model platforms.

Variable	Total (*n* = 200)	ChatGPT 4o (*n* = 20)	ChatGPT 5.1-Thinking (*n* = 20)	ChatGPT 5.2-Thinking (*n* = 20)	DeepSeek-R1 (*n* = 20)	Doubao (*n* = 20)	ERNIE Bot 5.0 Preview (*n* = 20)	Kimi K2 (*n* = 20)	Qwen3 (*n* = 20)	Qwen3-Max (*n* = 20)	Qwen3-Max-Thinking-Preview (*n* = 20)	*p* value
Words	274.50 (203.75, 378.75)	232.00 (214.75, 250.50)	238.00 (134.75, 322.50)	165.00 (84.50, 291.00)	360.00 (285.00, 490.75)	342.50 (290.75, 418.00)	267.50 (209.50, 511.50)	232.50 (182.75, 328.75)	458.50 (415.50, 602.50)	232.50 (206.75, 313.75)	236.50 (199.00, 301.00)	< 0.001
Sentences	16.00 (10.75, 24.25)	12.00 (10.00, 13.25)	19.50 (12.25, 31.00)	10.00 (4.75, 21.25)	22.00 (17.50, 32.25)	14.50 (13.00, 19.00)	11.50 (8.00, 24.00)	20.50 (14.75, 27.00)	29.50 (23.75, 39.50)	16.00 (10.00, 22.00)	15.00 (8.75, 22.25)	< 0.001
Syllables	519.00 (377.25, 732.00)	441.00 (398.00, 475.75)	460.00 (207.00, 617.25)	321.00 (157.75, 531.50)	653.00 (515.00, 940.00)	642.50 (551.50, 839.00)	499.00 (378.75, 908.75)	469.50 (341.00, 631.50)	915.50 (822.00, 1229.00)	449.50 (358.00, 575.00)	475.00 (394.50, 592.75)	< 0.001
ARI	15.59 (13.65, 17.52)	15.62 (13.80, 17.21)	12.22 (10.63, 13.14)	14.93 (13.58, 16.48)	15.86 (14.10, 16.80)	19.23 (17.65, 20.69)	17.19 (15.67, 18.09)	15.87 (14.11, 16.86)	15.29 (14.37, 16.67)	15.14 (13.53, 16.23)	15.25 (14.28, 17.76)	< 0.001
FRES	24.00 (18.00, 34.00)	26.50 (18.25, 32.00)	36.50 (27.75, 42.25)	27.50 (20.00, 35.00)	30.50 (19.00, 35.00)	19.50 (16.00, 26.00)	25.50 (15.00, 29.75)	19.50 (15.75, 31.00)	20.50 (17.75, 29.25)	21.50 (17.75, 32.50)	19.00 (14.75, 30.25)	0.003
GFOG	15.40 (13.78, 16.83)	15.20 (14.47, 17.12)	12.75 (11.70, 13.90)	14.90 (13.38, 15.83)	15.25 (13.20, 16.75)	16.85 (15.73, 17.90)	15.35 (14.10, 16.95)	16.00 (14.10, 16.80)	16.15 (14.40, 17.15)	15.60 (14.78, 16.33)	16.00 (14.47, 17.05)	< 0.001
FKGL	14.12 (12.68, 15.57)	14.47 (13.42, 16.22)	11.60 (10.21, 12.95)	13.78 (12.49, 14.97)	13.27 (12.14, 15.03)	16.48 (15.34, 17.07)	15.55 (14.86, 17.08)	13.80 (12.04, 14.69)	13.98 (12.86, 15.12)	13.69 (12.88, 14.61)	14.03 (13.16, 14.92)	< 0.001
CLI	15.80 (14.44, 17.29)	15.37 (14.05, 16.96)	13.97 (12.85, 14.98)	14.84 (14.01, 16.93)	15.47 (14.12, 16.22)	16.23 (14.79, 17.14)	14.77 (14.29, 16.53)	17.33 (15.49, 18.45)	16.94 (15.76, 17.52)	16.80 (15.59, 17.92)	17.13 (15.47, 17.91)	< 0.001
SMOG	12.30 (10.84, 13.87)	13.44 (11.92, 14.14)	10.18 (8.80, 11.07)	12.00 (10.62, 13.06)	11.98 (10.92, 13.25)	14.63 (13.87, 15.12)	14.06 (13.15, 14.92)	11.22 (10.48, 12.99)	11.95 (11.22, 13.37)	11.91 (10.75, 12.76)	12.42 (11.05, 13.73)	< 0.001
LW	15.52 (13.08, 18.13)	14.41 (12.07, 15.78)	15.18 (12.38, 18.51)	15.08 (12.58, 18.54)	13.71 (12.76, 15.09)	16.34 (15.31, 17.68)	16.25 (15.36, 19.78)	19.66 (15.68, 31.08)	14.14 (12.12, 16.02)	16.31 (13.73, 20.79)	16.45 (12.97, 19.71)	< 0.001

Values are presented as median (Q1, Q3). Omnibus *p* values were obtained using the Kruskal–Wallis H test. ARI, Automated Readability Index; FRES, Flesch Reading Ease Score; GFOG, Gunning Fog Index; FKGL, Flesch–Kincaid Grade Level; CLI, Coleman–Liau Index; SMOG, Simple Measure of Gobbledygook; LW, Linsear Write Formula.

#### Topic-specific characteristics of diabetes education texts generated by large language model platforms

In the topic-stratified analyses (Table 4), PEMAT-P understandability did not differ significantly across topics (*p* = 0.356), and neither the PEMAT-P total score nor the GQS showed a significant topic effect (*p* = 0.247 and *p* = 0.182, respectively). By contrast, PEMAT-P actionability differed significantly across topics (*χ*^2^ = 42.74, *p* < 0.001), with lower scores for “Fundamental Understanding of the Disease” and “Treatment and Technology”. EQIP-36 also varied significantly by topic (*χ*^2^ = 27.91, *p* < 0.001), with the lowest scores observed for “Fundamental Understanding of the Disease”. Text-length metrics, including word count, sentence count, and syllable count, differed significantly across topics (*p* = 0.027, *p* = 0.011, and *p* = 0.002, respectively). Among the readability measures, FRES, GFOG, CLI, SMOG, and LW differed significantly across topics, whereas ARI and FKGL did not.

**Table 4:** Topic-specific characteristics of diabetes education texts generated by large language model platforms.

Variable	Total (*n* = 200)	Complications, psychosocial care, and prognosis (*n* = 40)	Daily management and prevention (*n* = 40)	Diagnosis and classification (*n* = 40)	Fundamental understanding of the disease (*n* = 40)	Treatment and technology (*n* = 40)	*p* value
PEMAT-P understandability	76.90 (66.70, 84.60)	76.90 (69.20, 78.50)	69.20 (63.08, 78.83)	78.45 (69.20, 85.95)	75.00 (66.70, 84.60)	76.90 (66.70, 84.60)	0.356
PEMAT-P actionability	20.00 (0.00, 40.00)	20.00 (20.00, 40.00)	20.00 (0.00, 60.00)	20.00 (0.00, 40.00)	0.00 (0.00, 20.00)	0.00 (0.00, 0.00)	< 0.001
PEMAT-P total score	58.80 (50.00, 68.20)	61.10 (55.60, 67.08)	58.80 (50.00, 77.80)	64.10 (54.20, 72.20)	54.45 (49.27, 65.20)	57.20 (50.00, 61.10)	0.247
EQIP-36 score	38.20 (32.90, 43.90)	41.20 (36.39, 45.38)	38.20 (34.70, 50.35)	39.85 (36.12, 43.82)	30.00 (24.80, 38.03)	37.48 (32.53, 41.23)	< 0.001
Words	274.50 (203.75, 378.75)	285.50 (211.25, 377.75)	228.50 (181.00, 312.50)	267.50 (192.00, 386.50)	250.50 (176.75, 369.75)	320.00 (253.00, 429.25)	0.027
Sentences	16.00 (10.75, 24.25)	19.50 (12.75, 30.00)	12.00 (8.00, 16.50)	17.50 (12.75, 24.50)	14.50 (10.75, 24.00)	21.00 (13.50, 25.50)	0.011
Syllables	519.00 (377.25, 732.00)	557.00 (425.75, 739.50)	429.50 (311.75, 571.75)	469.00 (340.00, 678.75)	484.50 (344.50, 765.75)	636.00 (514.00, 881.75)	0.002
ARI	15.59 (13.65, 17.52)	15.23 (13.62, 18.71)	15.88 (13.56, 17.88)	15.27 (13.10, 16.73)	15.45 (13.61, 17.17)	15.89 (15.13, 17.75)	0.238
FRES	24.00 (18.00, 34.00)	22.00 (18.00, 30.25)	34.50 (20.00, 42.25)	29.00 (19.75, 40.75)	20.50 (16.00, 28.00)	20.00 (16.75, 29.00)	< 0.001
GFOG	15.40 (13.78, 16.83)	15.40 (14.00, 16.33)	13.35 (11.78, 15.50)	14.50 (12.93, 16.43)	16.55 (15.28, 17.15)	16.20 (15.30, 17.30)	< 0.001
FKGL	14.12 (12.68, 15.57)	13.78 (12.88, 16.29)	13.54 (11.74, 15.58)	13.87 (11.33, 14.83)	14.70 (13.15, 15.61)	14.56 (13.41, 16.16)	0.101
CLI	15.80 (14.44, 17.29)	15.92 (14.77, 17.13)	15.50 (13.67, 17.22)	15.28 (13.98, 16.94)	15.68 (14.16, 16.92)	16.61 (15.41, 17.69)	0.022
SMOG	12.30 (10.84, 13.87)	11.87 (10.78, 14.24)	11.57 (10.11, 13.54)	11.71 (10.49, 13.37)	13.02 (11.73, 14.21)	12.77 (11.62, 14.26)	0.011
LW	15.52 (13.08, 18.13)	17.00 (13.95, 18.88)	15.20 (12.43, 17.94)	13.50 (12.10, 15.53)	15.48 (14.29, 16.89)	16.70 (14.89, 22.02)	< 0.001
GQS score	4.00 (4.00, 4.00)	4.00 (4.00, 5.00)	4.00 (4.00, 5.00)	4.00 (4.00, 4.00)	4.00 (4.00, 4.00)	4.00 (4.00, 4.00)	0.182

Values are presented as median (Q1, Q3). Omnibus P values were obtained using the Kruskal–Wallis H test. PEMAT-P, Patient Education Materials Assessment Tool for Printable Materials; EQIP-36, Ensuring Quality Information for Patients, 36-item instrument; GQS, Global Quality Score; ARI, Automated Readability Index; FRES, Flesch Reading Ease Score; GFOG, Gunning Fog Index; FKGL, Flesch–Kincaid Grade Level; CLI, Coleman–Liau Index; SMOG, Simple Measure of Gobbledygook; LW, Linsear Write Formula.

## Text quality

### Text quality across large language model platforms

**Figure 1:**
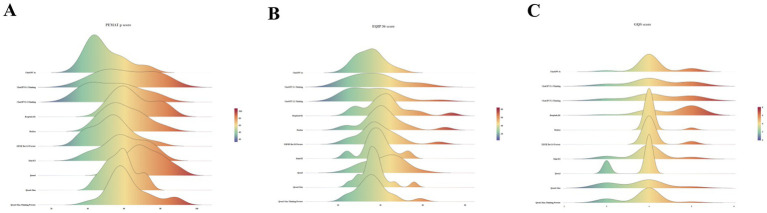
Distributions of diabetes education texts generated by different platforms in terms of **(A)** PEMAT-P total score, **(B)** EQIP-36, and **(C)** GQS.

The ridgeline plot (Figure 1) illustrates platform-specific differences in the distributions of the PEMAT-P total score, EQIP-36, and GQS. For the PEMAT-P total score, ChatGPT 4o showed a left-shifted and relatively concentrated distribution, whereas ChatGPT 5.1-Thinking and ChatGPT 5.2-Thinking were shifted to the right and displayed a pronounced high-score tail. Qwen3 was the most right-shifted overall, consistent with greater suitability for patient education; however, Qwen3-Max and Qwen3-Max-Thinking-Preview showed broader, bimodal distributions, which may indicate greater output variability. For EQIP-36, the density peaks for most platforms clustered between 30 and 45, and only a few platforms, such as Doubao and ERNIE Bot 5.0 Preview, exhibited a long right tail, suggesting that higher information-quality scores were driven by a limited subset of outputs rather than by uniformly improved performance across all texts. For GQS, most texts clustered around a score of 4, whereas DeepSeek-R1 and the later ChatGPT versions showed heavier right tails, indicating a greater likelihood of generating higher-quality texts. By contrast, Kimi K2 showed a narrower distribution, consistent with greater output consistency but less extension at the upper end of the score range. Taken together, these patterns indicate both between-platform differences and within-platform variability in AI-generated DSMES texts, with such within-platform variability potentially contributing to inconsistency in actionable recommendations.

### Text quality across topic dimensions

**Figure 2:**
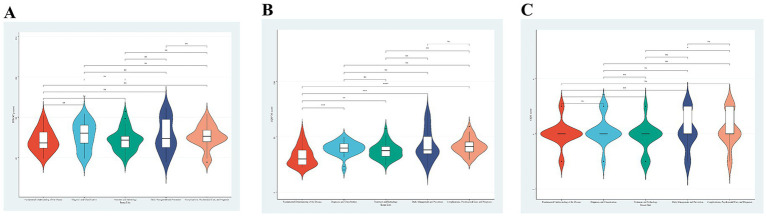
Distributions of diabetes education texts across topic dimensions for **(A)** PEMAT-P total score, **(B)** EQIP-36, and **(C)** GQS.

The violin plot (Figure 2) illustrates heterogeneity in text-quality distributions across topic domains. The PEMAT-P total score showed substantial overlap across domains and did not differ significantly overall (*χ*^2^ = 5.42, *p* = 0.247). Median PEMAT-P scores were relatively higher for “Diagnosis and Classification” [64.10 (54.20–72.20)] and lowest for “Fundamental Understanding of the Disease” [54.45 (49.27–65.20)]. By contrast, EQIP-36 differed significantly across domains (*χ*^2^ = 27.91, *p* < 0.001). Scores were lower for “Fundamental Understanding of the Disease” [30.00 (24.80–38.03)] and higher for “Complications, Psychosocial Care, and Prognosis” [41.20 (36.39–45.38)], with the remaining domains showing intermediate values. The “Daily Management and Prevention” domain showed a broader distribution, consistent with greater variability in information quality. GQS clustered around 4 and did not differ significantly across domains (*χ*^2^ = 6.24, *p* = 0.182), although longer high-score tails were observed for “Complications, Psychosocial Care, and Prognosis” and “Daily Management and Prevention.”

### Text readability

**Figure 3:**
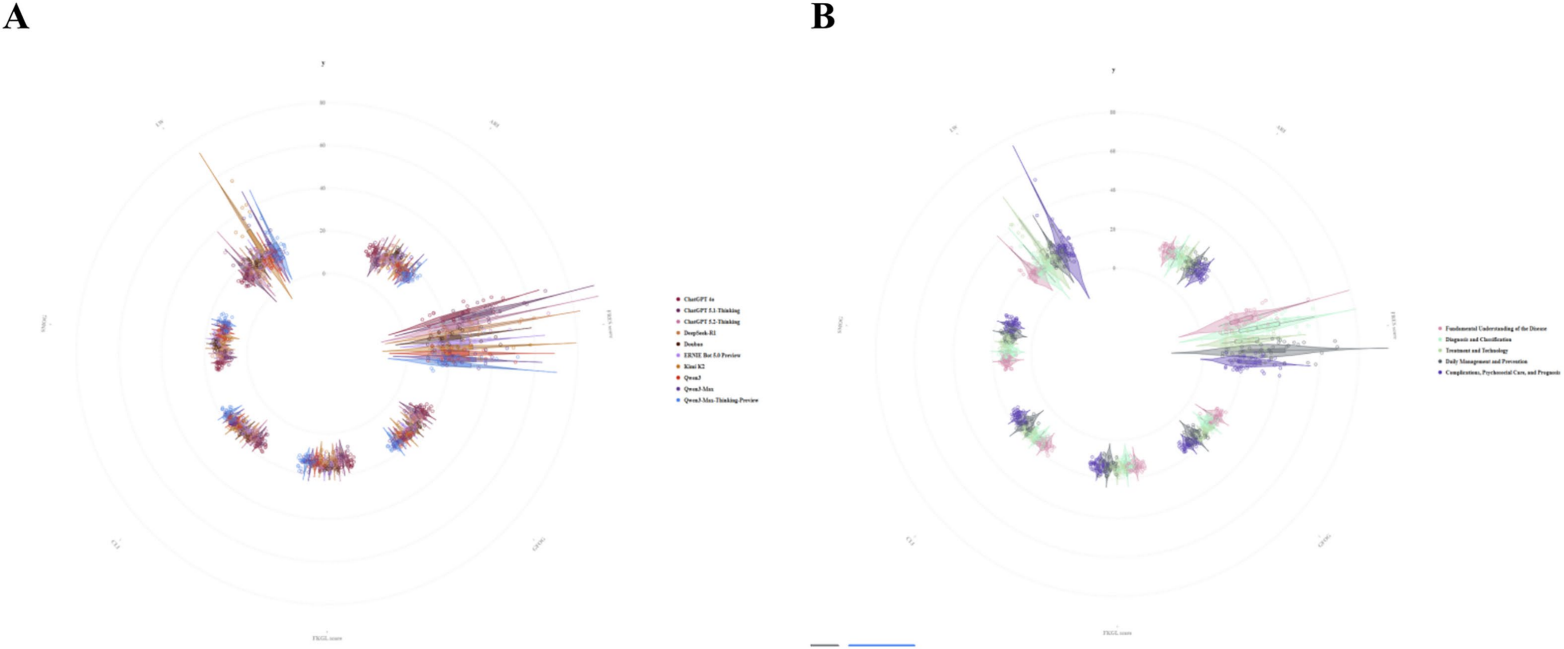
Distributions of readability metrics for diabetes education materials across **(A)** LLM platforms and **(B)** readability dimensions.

Polar-coordinate violin plots (Figure 3) showed substantial variation in the readability of DSMES materials generated by different LLMs. This variation was most pronounced for FRES, which showed the greatest between-model separation and dispersion. ChatGPT 4o and ChatGPT 5.1-Thinking/ChatGPT 5.2-Thinking exhibited higher central values for FRES, indicating generally more readable text. By contrast, Qwen3-Max and Qwen3-Max-Thinking-Preview showed lower central values and longer tails for LW, suggesting a higher prevalence of long words and more complex sentence construction. DeepSeek-R1, Doubao, ERNIE Bot 5.0 Preview, Kimi K2, and Qwen3 showed intermediate patterns. In comparison, the grade-based complexity indices—ARI, FKGL, GFOG, CLI, and SMOG—showed substantial overlap across models, indicating that the between-model differences were concentrated in overall readability and long-word complexity rather than reflecting a uniform shift across all complexity metrics.

After stratification by topic, FRES was higher, indicating better readability, for Fundamental Understanding of the Disease and Diagnosis and Classification. Materials addressing Complications, Psychosocial Care, and Prognosis showed lower FRES values and greater variability in LW, consistent with denser terminology and more complex expression. Daily Management and Prevention, as well as Treatment and Technology, showed intermediate patterns. Overall, the readability of LLM-generated DSMES materials varied across platforms and topics; for high-complexity topics, broader information coverage may be accompanied by a greater reading burden, which could in turn hinder patient understanding and the translation of knowledge into self-management behaviours.

### Correlation analysis

#### Within-platform internal correlations

**Figure 4:**
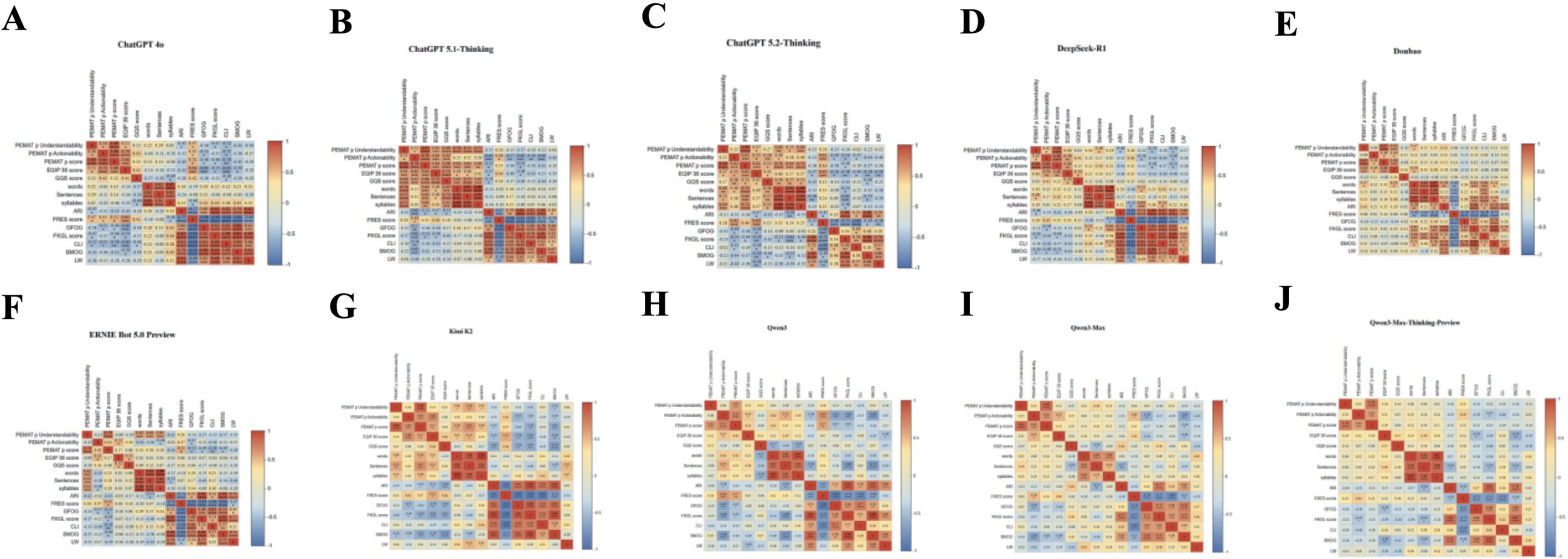
Correlations among actionability, information quality, overall quality, and readability metrics for diabetes education texts across platforms. **(A)** ChatGPT 4o, **(B)** ChatGPT 5.1-Thinking, **(C)** ChatGPT 5.2-Thinking, **(D)** DeepSeek-R1, **(E)** Doubao, **(F)** ERNIE Bot 5.0 Preview, **(G)** Kimi K2, **(H)** Qwen3, **(I)** Qwen3-Max, and **(J)** Qwen3-Max-Thinking-Preview.

The correlation heatmap (Figure 4) showed a broadly similar within-model correlation structure across LLMs. PEMAT-P understandability, actionability, and total score were positively correlated. EQIP-36 was moderately to strongly correlated with PEMAT-P, and GQS showed moderate correlations with both. Length-related metrics, including word count, sentence count, and syllable count, were strongly intercorrelated. Readability indices, including ARI, FKGL, CLI, SMOG, GFOG, and LW, were positively correlated with one another, whereas FRES was strongly negatively correlated with these grade-level and complexity-based measures, indicating substantial collinearity among readability metrics. In ChatGPT 4o, FRES was correlated with EQIP-36, GQS, and PEMAT-P (*r* = 0.42–0.64), whereas CLI and FKGL were negatively associated with PEMAT-P and EQIP-36 (*r* = −0.35 to −0.65). Across models, higher quality and actionability generally coincided with lower complexity, although the magnitude of these correlations varied. For example, quality–readability correlations were close to zero in Doubao and Qwen3-Max, and the correlation between PEMAT-P understandability and actionability was weaker in ERNIE Bot 5.0 Preview and Kimi K2. Overall, AI-generated DSMES materials showed inconsistent associations among quality, readability, and actionable instructions. Without control of target reading level and the use of structured action templates, reading burden and implementability may vary substantially, potentially reducing the effectiveness of health education.

#### Within-topic correlations among variables across dimensions

**Figure 5:**
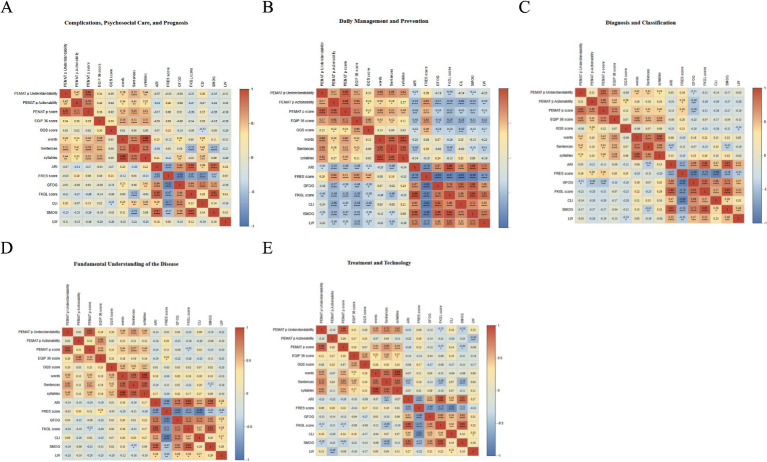
Correlation analyses of diabetes education texts across dimensions, including actionability, information quality, overall quality, and readability metrics. **(A)** Complications, Psychosocial Care, and Prognosis; **(B)** Daily Management and Prevention; **(C)** Diagnosis and Classification; **(D)** Fundamental Understanding of the Disease; and **(E)** Treatment and Technology.

Topic-specific correlation heatmaps (Figures 5A–E) showed an overall stable correlation structure, together with topic-specific variation in the associations among quality, readability, and actionability. Across the five topics, PEMAT-P understandability was strongly correlated with the PEMAT-P total score (*r* = 0.83–0.91), and PEMAT-P actionability was moderately to strongly correlated with the total score (Complications, Psychosocial Care, and Prognosis, *r* = 0.74; Daily Management and Prevention, *r* = 0.86; Diagnosis and Classification, *r* = 0.69). Length-related metrics, including word count, sentence count, and syllable count, were highly correlated within topics (word count vs. syllable count: Complications, Psychosocial Care, and Prognosis, *r* = 0.99; Daily Management and Prevention, *r* = 0.97). Readability grade-level and complexity indices (ARI, GFOG, FKGL, CLI, SMOG, and LW) were strongly intercorrelated and inversely correlated with FRES (Daily Management and Prevention: FRES–GFOG, *r* = −0.93; FRES–CLI, *r* = −0.91; Diagnosis and Classification: both *r* = −0.88), indicating substantial collinearity. Associations between quality and readability varied by topic. In Daily Management and Prevention, FRES was positively correlated with EQIP-36, PEMAT-P actionability, and GQS (*r* = 0.65, 0.64, and 0.49, respectively), whereas EQIP-36 showed stronger negative correlations with complexity indices (EQIP-36–CLI, *r* = −0.58; EQIP-36–FKGL, *r* = −0.59). By contrast, in Complications, Psychosocial Care, and Prognosis, associations with FRES were close to null (PEMAT-P understandability–FRES, *r* = −0.02; EQIP-36–FRES, *r* = 0.06). Overall, higher quality and actionability were not consistently accompanied by a lower reading burden; in high-complexity topics, LLM-generated texts may achieve high scores whilst remaining difficult to understand or implement.

#### Within-domain correlations among variables

**Figure 6:**
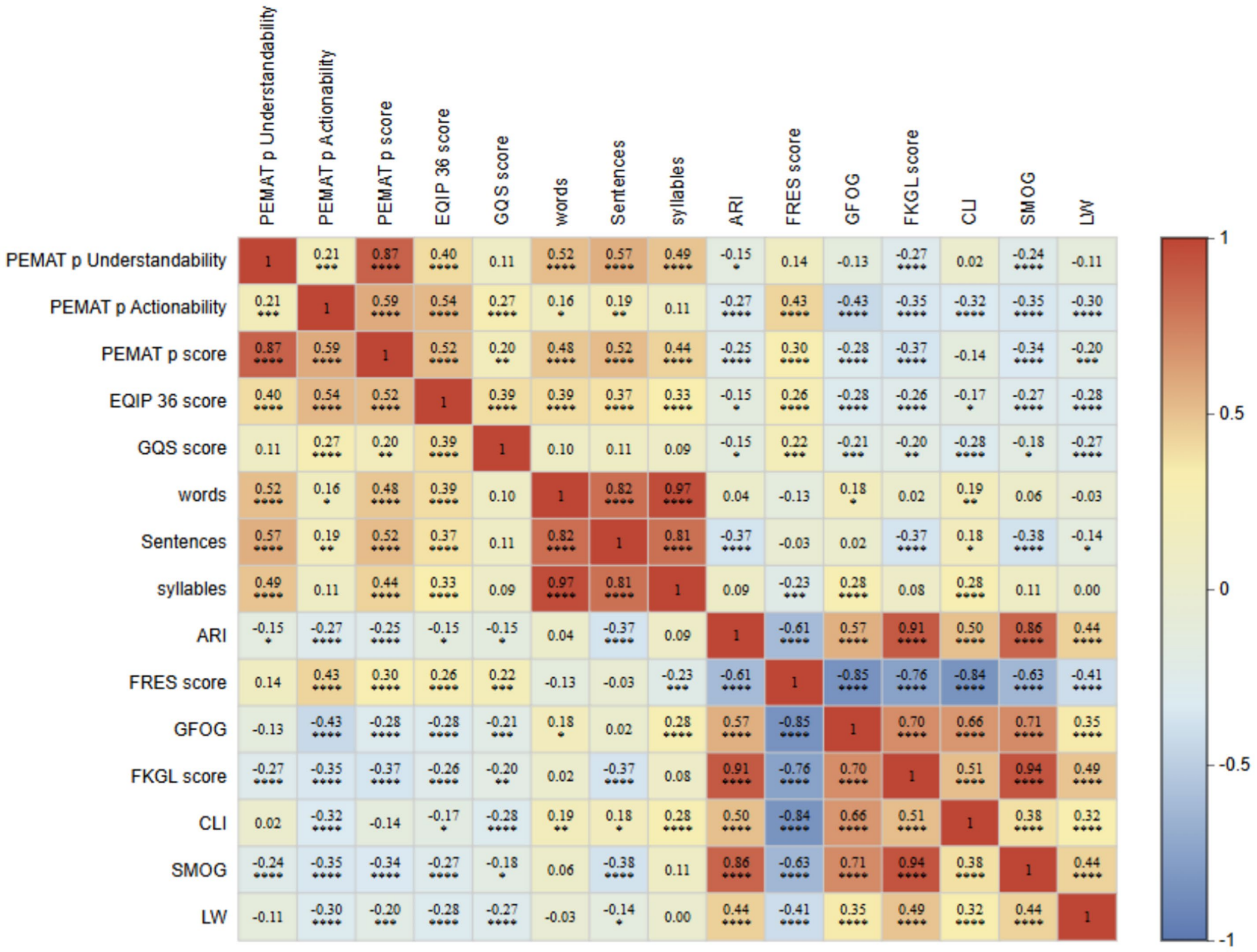
Correlation matrix (heatmap) of actionability, information quality/overall quality, and readability metrics for AI-generated diabetes education texts.

The correlation heatmap (Figure 6) identified three clusters: (i) quality and actionability, (ii) text length, and (iii) readability complexity. Within the quality-and-actionability cluster, the PEMAT-P total score was strongly correlated with understandability (*r* = 0.87) and moderately correlated with actionability (*r* = 0.59). EQIP-36 was correlated with the PEMAT-P total score (*r* = 0.52) and actionability (*r* = 0.54), whereas GQS was moderately correlated with EQIP-36 (*r* = 0.39) but only weakly correlated with PEMAT-P (*r* = 0.11–0.27). Text-length measures were highly collinear (word count–syllable count, *r* = 0.97; word count–sentence count, *r* = 0.82) and showed moderate positive correlations with understandability (word count–PEMAT-P understandability, *r* = 0.52; sentence count–PEMAT-P understandability, *r* = 0.57). Readability indices showed the strongest collinearity: ARI was correlated with FKGL (*r* = 0.91) and SMOG (*r* = 0.86), and FKGL was correlated with SMOG (*r* = 0.94). FRES was strongly negatively correlated with GFOG, CLI, FKGL, and ARI (*r* = −0.85, −0.84, −0.76, and −0.61, respectively).

Across clusters, actionability appeared to align more closely with readability than did understandability. FRES was positively correlated with actionability (*r* = 0.43), whereas readability-complexity indices, including GFOG, FKGL, CLI, SMOG, and LW, were negatively correlated with actionability (*r* = −0.30 to −0.43). By contrast, understandability was only weakly correlated with FRES (*r* = 0.14), suggesting that actionability may track linguistic simplification more closely, whereas understandability may be partly related to text length. Increasing coverage or length alone may therefore not improve implementability. Cross-platform generation should incorporate target grade-level constraints and structured, stepwise action templates to reduce cognitive burden and standardise action instructions.

### Exploratory regression analyses

Exploratory stepwise multivariable linear regression models were fitted to identify readability-related correlates of patient-education quality outcomes. ARI, FRES, GFOG, FKGL, CLI, SMOG, and LW were entered as candidate variables, and the outcomes were the PEMAT-P total score, EQIP-36 total score, and GQS. Because these readability indices are conceptually related and may exhibit substantial multicollinearity, the models were considered exploratory rather than confirmatory. Accordingly, variables retained in these exploratory models should be interpreted as candidate readability-related correlates of quality outcomes rather than definitive independent predictors. VIFs were reported, and coefficient directions were interpreted cautiously.

### Readability-related correlates of PEMAT-P total score

In the exploratory stepwise model with the PEMAT-P total score as the dependent variable, FKGL and ARI were retained (Table 5) and together explained 21.1% of the variance in the PEMAT-P total score (*R*^2^ = 0.211; adjusted *R*^2^ = 0.203). FKGL was inversely associated with the PEMAT-P total score (*B* = −4.682, *p* < 0.001), indicating that materials requiring a higher grade-level reading ability tended to receive lower PEMAT-P ratings. Within this exploratory model, ARI was positively associated with the PEMAT-P total score (*B* = 2.399, *p* < 0.001). However, because the VIFs for the two retained variables were approximately 5.81, indicating moderate-to-high multicollinearity and potential suppression effects, these coefficients should be interpreted with caution. Overall, the model suggests that a greater reading burden may be associated with poorer PEMAT-P performance, although the direction and magnitude of the individual coefficients should not be overinterpreted.

**Table 5:** Exploratory stepwise multivariable linear regression identifying readability-related correlates of PEMAT-P total score.

Predictor	Unstandardised coefficients		Standardised coefficients (β)	*t*	*p*	Collinearity diagnostics	
	*B*	Std. Error				VIF	Tolerance
Constant	87.468**	4.805	–	18.202	*p* < 0.001	–	–
FKGL	−4.682**	0.807	−0.885	−5.800	*p* < 0.001	5.807	0.172
ARI	2.399**	0.691	0.529	3.469	*p* < 0.001	5.807	0.172
*R* ^2^	0.211						
Adjusted *R*^2^	0.203						
*F*	*F* (2.0,197.0) = 26.315, *p* < 0.001						
Durbin–Watson	1.510						

Variable = PEMAT-P score. *p < 0.05; **p < 0.01.

Candidate predictors included ARI, FRES, GFOG, FKGL, CLI, SMOG, and LW; the exploratory model selected FKGL and ARI.

### Readability-related correlates of EQIP-36 total score

In the exploratory stepwise model with the EQIP-36 total score as the dependent variable, only FRES was retained (Table 6). The model explained 16.5% of the variance (*R*^2^ = 0.165; adjusted *R*^2^ = 0.161). Higher FRES values, indicating greater reading ease, were associated with higher EQIP-36 total scores (*B* = 0.320, *p* < 0.001), suggesting better-rated information quality and presentation. This finding indicates an exploratory association between greater reading ease and higher information quality; however, it should not be interpreted as evidence of an independent causal effect.

**Table 6:** Exploratory stepwise multivariable linear regression identifying readability-related correlates of EQIP-36 total score.

Predictor	Unstandardised Coefficients		Standardised Coefficients (*β*)	*t*	*p*	Collinearity diagnostics	
	*B*	Std. Error				VIF	Tolerance
Constant	30.014**	1.498	–	20.030	p < 0.001	–	–
FRES	0.320**	0.051	0.407	6.262	p < 0.001	1.000	1.000
*R* ^2^	0.165						
Adjusted *R*^2^	0.161						
*F*	*F* (1.0,198.0) = 39.216, *p* < 0.001						
Durbin–Watson	1.780						

Dependent variable = EQIP-36 score. *p < 0.05; **p < 0.01.

The exploratory model selected FRES.

### Readability-related correlates of GQS

In an exploratory stepwise regression model with GQS as the dependent variable, CLI and LW were retained in the final model (Table 7). The model accounted for 12.0% of the variance in GQS (*R*^2^ = 0.120; adjusted *R*^2^ = 0.111). CLI was inversely associated with GQS (*B* = −0.072, *p* < 0.001), and LW was likewise inversely associated with GQS (*B* = −0.014, *p* = 0.025), suggesting that greater reading difficulty was associated with lower overall quality. Given the intercorrelations among readability indices and the exploratory nature of the analysis, these retained variables should be interpreted as candidate correlates of lower GQS rather than definitive independent predictors.

**Table 7:** Exploratory stepwise multivariable linear regression identifying readability-related correlates of GQS.

Predictor	Unstandardised coefficients		Standardised Coefficients (β)	*t*	*p*	Collinearity diagnostics	
	*B*	Std. Error				VIF	Tolerance
Constant	5.415**	0.296	–	18.313	*p* < 0.001	–	–
CLI	−0.072**	0.020	−0.257	−3.614	*p* < 0.001	1.132	0.883
LW	−0.014*	0.006	−0.161	−2.264	0.025	1.132	0.883
*R* ^2^	0.120						
Adjusted *R*^2^	0.111						
*F*	*F* (2.0,197.0) = 13.461, *p* < 0.001						
Durbin–Watson	1.578						

Dependent variable = GQS score. *p < 0.05; **p < 0.01.

The exploratory model selected CLI and LW.

## Discussion

### Key findings

This study compared the quality of DSMES texts generated by 10 LLMs. Marked between-platform heterogeneity was observed for GQS and for the PEMAT-P understandability and total scores (all *p* < 0.001), whereas EQIP-36 did not differ significantly overall (*p* = 0.062). Platforms also differed significantly in text length and in multiple readability metrics, consistent with previous reports showing that medical dialogue model outputs are often of generally high quality, yet remain variable and frequently linguistically complex (36, 37)^.^

DeepSeek-R1 achieved the highest median GQS (5.00). Higher EQIP-36 scores were observed primarily for DeepSeek-R1, Qwen3, and Doubao, and these models also tended to generate longer texts overall; Qwen3 had the highest median word count. By contrast, ChatGPT 5.1-Thinking showed more favourable readability, as reflected by higher FRES and lower FKGL values, but only intermediate EQIP-36 performance. Doubao and ERNIE Bot 5.0 Preview showed higher grade-level metrics, including FKGL, suggesting denser terminology and greater syntactic complexity.

These differences may reflect the combined effects of training data, model architecture, and the breadth of medical knowledge represented in each model. In our evaluation, no single model performed best across all metrics. Accordingly, when LLMs are used to generate patient-education materials, health-care institutions should balance content depth against understandability and select platforms according to the needs of the target population. The observed cross-platform inconsistency further supports the need for standardised quality and readability criteria to ensure minimum performance standards across sources and to reduce the influence of platform choice on patient-education outcomes.

### Effects of diabetes education content dimensions on text characteristics

Diabetes patient education encompasses multiple content dimensions, including Fundamental Understanding of the Disease, Diagnosis and Classification, Treatment and Technology, Daily Management and Prevention, and Complications, Psychosocial Care, and Prognosis. We observed topic-specific differences in both readability and information completeness. Among the readability metrics, FRES showed the greatest variation across topics (*p* < 0.001): “Daily Management and Prevention” had the highest FRES, whereas “Treatment and Technology” and “Fundamental Understanding of the Disease” had lower FRES values and higher SMOG scores (SMOG, *p* = 0.011). These findings are consistent with previous research in health communication showing that more technical topics are more difficult to communicate in plain language and that dense medical terminology may exceed patients’ health literacy levels; substantial variation in readability across diseases and topics has also been reported (38).

Information completeness, as assessed by EQIP-36, also differed significantly across topics (*p* < 0.001). “Fundamental Understanding of the Disease” had the lowest score, indicating a greater risk of incomplete coverage of essential information, whereas “Complications, Psychosocial Care, and Prognosis” scored relatively higher. These findings support the use of topic-specific prompt optimisation and human review when LLMs are used to generate patient education materials, in order to balance readability with adequate coverage of essential medical information. Topic-level assessment may help identify higher-risk areas characterised by poorer readability or missing content and thereby inform targeted quality improvement.

### Actionability and patient education effectiveness

Clear and complete action-oriented instructions are essential if patient-education materials are to support behaviour change. According to PEMAT-P, diabetes education texts generated across platforms showed generally low actionability. Although understandability scores were relatively high (mean close to 70%), actionability scores were lower and often lacked concrete, implementable steps. Actionability also did not differ significantly across platforms (*p* = 0.313), suggesting a shared limitation across models rather than a platform-specific deficiency. This finding is consistent with previous evidence that online patient-education resources often provide insufficient actionable information (39).

We further observed that some LLM-generated texts were rich in information but lacked practical guidance, which may limit patients’ ability to translate knowledge into practise. Effective materials should present self-care guidance as stepwise actions, often using action verbs, and, where feasible, pair instructions with demonstrations or supportive tools. In the PEMAT development study, Shoemaker and colleagues reported that materials with higher actionability also received higher understandability and actionability ratings (26). Similar gaps have been documented in conventional resources; in Lipari and colleagues’ evaluation of 13 online diabetes materials, only one met actionability standards (39).

Overall, LLMs readily generate explanatory content but may underprovide the operational detail required for implementation. Prompts that explicitly request step-by-step instructions, together with clinician review to add key operational elements, may improve both actionability and clinical usability.

### Balancing readability and clinical applicability

Our results indicate a trade-off between readability and the completeness of clinically relevant information in patient education materials. When written materials exceed a patient’s health literacy level, the information provided may be difficult to understand and apply in practise (40, 41). In the general US population, the average reading level is approximately at the eighth-grade level, and organisations involved in patient care typically recommend that patient-facing materials be written at the fifth- to sixth-grade level (42–45). Poor readability increases the risk of misunderstanding and may contribute to ineffective health behaviours, thereby increasing the likelihood of adverse health outcomes. In one study, the reading level of diabetic foot care materials was reduced from grade 11 to grade 6, and the proportion of students who were able to understand the content independently increased from less than 20% to more than 60% (44). This improvement suggests that reducing linguistic barriers can substantially enhance patients’ uptake of important information. Similar findings were reported by Rouhi et al. (46), who found that artificial intelligence improved the readability of patient education materials on aortic stenosis, although the generated texts did not consistently achieve the recommended sixth-grade reading level.

On the other hand, excessive simplification may omit important details and supporting evidence, thereby undermining clinical applicability and validity; high-quality materials should strike a balance between plain language and scientific accuracy (47–49). We found that unfiltered LLM outputs may fall disproportionately on either side of the spectrum between information overload and colloquial oversimplification, highlighting the need for human evaluation within a health communication framework. A recent cross-platform study of patient education materials for chronic heart failure similarly showed that generative AI outputs may face challenges in balancing content quality, readability, and actionability across platforms (50). Recent literature indicates that GPT-4 can reduce reading grade levels by approximately two grades without compromising accuracy or content completeness (51). Ayre et al. (52) also showed that ChatGPT can, with expert guidance, rephrase complex medical text into plain language without loss of key content.

Research on chronic disease self-management further suggests that understanding promotes implementation, which in turn reinforces understanding and improves health-related behaviours (53–55). The Technology Acceptance Model (TAM) proposes that acceptance of AI-generated materials is influenced by perceived usefulness and perceived ease of use (56–58). In other words, materials that are difficult to understand or act upon may erode trust and reduce sustained use (59, 60). Accordingly, a human–AI workflow, in which evidence is translated into language that is comprehensible to patients whilst preserving actionable guidance, may provide a more balanced, readable, and informative approach to evidence translation and may also support long-term self-management.

## Limitations and future directions

This study has several limitations. First, although we conducted a cross-platform comparison of diabetes education texts generated by 10 LLMs using a multi-metric framework assessing quality, readability, and actionability, the evaluation was limited to English-language outputs. The findings may therefore not be generalisable to patient-education materials produced in other languages or applied in different cultural and health-literacy contexts. Second, to improve cross-platform comparability, we used a standardised prompt and restricted generation to a single-turn response format. Consequently, we did not examine whether alternative prompting strategies, target grade-level instructions, multi-turn interactions, or personalised contextual inputs could improve readability, completeness, or actionability. Third, the analyses focused on textual characteristics and instrument-based quality assessments rather than real-world patient use. Although PEMAT-P, EQIP-36, GQS, and readability indices provide useful proxy measures, they do not directly capture patient comprehension, behavioural uptake, self-management performance, or clinical outcomes. Accordingly, the real-world effectiveness of these materials in diabetes education remains to be established. Fourth, this study provides a time-specific cross-sectional assessment of model outputs. Because LLM versions are updated frequently, response quality, readability, and safety profiles may change over time; ongoing monitoring is therefore needed to assess temporal stability and content drift. Fifth, the exploratory regression analyses did not include platform or model as covariates, and the readability indices showed substantial conceptual overlap. The regression coefficients should therefore be interpreted as reflecting overall covariation rather than definitive platform-specific independent effects. Future studies may benefit from theory-driven variable selection and mixed-effects modelling to further test the robustness of these associations.

Future work should incorporate real-world usability testing involving patients and nursing staff across outpatient nursing clinics, health management programmes, and community education settings, and should evaluate educational effectiveness using behavioural and health outcome measures. Comparative studies are also needed to assess structured prompting, stepwise action-instruction templates, retrieval-augmented generation, and human–AI collaborative review as strategies to reduce reading burden whilst preserving information completeness, safety, and appropriateness. Finally, a cross-platform quality-control and evaluation framework that can be updated sustainably is needed to keep pace with the rapid evolution of LLMs.

## Conclusion

We conducted a cross-platform quantitative comparison of diabetes self-management patient-education texts generated by 10 LLMs, evaluating GQS, PEMAT-P (understandability and actionability), EQIP-36, and multiple readability metrics. Significant between-platform differences were observed for GQS and PEMAT-P, indicating that model selection can influence content quality and the provision of actionable guidance. By contrast, differences in EQIP-36 were relatively limited, suggesting that standards of information presentation may depend more on writing conventions and generation templates.

In topic-stratified analyses, GQS, the PEMAT-P total score, and PEMAT-P understandability did not differ significantly across topic domains, whereas PEMAT-P actionability, EQIP-36, and several readability metrics varied by topic, indicating that topic complexity may shape the readability–usability profile of generated materials. Correlation analyses and exploratory regression models suggested that a greater reading burden tended to co-occur with lower quality, actionability, and information-presentation scores; however, these associations should be interpreted as exploratory patterns of overall covariation rather than stable independent effects.

Overall, LLMs can generate generally usable diabetes education texts, but substantial cross-platform heterogeneity remains in quality consistency, readability, and the completeness of action-oriented instructions. Clinical and public health implementation should therefore incorporate careful platform selection, structured prompting, human–AI review, and ongoing quality monitoring to support patient education that is safe, readable, and actionable.

## Data Availability

The original contributions presented in the study are included in the article/supplementary material, further inquiries can be directed to the corresponding authors.
